# Analysis of Skin Regeneration and Barrier-Improvement Efficacy of Polydeoxyribonucleotide Isolated from Panax Ginseng (C.A. Mey.) Adventitious Root

**DOI:** 10.3390/molecules28217240

**Published:** 2023-10-24

**Authors:** Kwang-Soo Lee, Soyeon Lee, Hyesoo Wang, Geonhee Lee, Seolyeong Kim, Yang-Hwan Ryu, Nicole Hyesoo Chang, Yong-Won Kang

**Affiliations:** 1 Bio Convergence Material Division, Biosolution Co., Ltd., Seoul 06746, Republic of Korea; hallym99@biosolutions.co.kr (K.-S.L.); lsy8769@biosolutions.co.kr (S.L.); brain1905@biosolutions.co.kr (Y.-H.R.); 2 Non-Clinical R&D Center, Biosolution Co., Ltd., Seoul 06746, Republic of Korea; dlrjsgml2014@biosolutions.co.kr (G.L.); tjfud2735@biosolutions.co.kr (S.K.); 3 Division of Biotechnology, College of Life Sciences & Biotechnology, Korea University, Seoul 02841, Republic of Korea; 4 Department of Medicine, McMaster University, Hamilton, ON L8S 4L8, Canada; ddcjs426@hotmail.com

**Keywords:** polydeoxyribonucleotide, plant-derived polydeoxyribonucleotide, Panax ginseng, skin regeneration, skin barrier, wound healing

## Abstract

Polydeoxyribonucleotide (PDRN) has the ability to regenerate skin cells and improve the skin barrier and wound healing. This study investigated the possibility of replacing animal-derived PDRN with plant-derived PDRN. To test this, the adventitious roots of Korean ginseng (Panax ginseng C.A. Meyer), which is commonly used to treat various diseases, were suspension-cultivated through tissue culture; subsequently, PDRN was purified using microfluidization, an ultra-high-pressure physical grinding method. The results showed that purified Panax PDRN was effective in healing skin wounds and enhancing the skin barrier. Panax PDRN promoted the proliferation of keratinocytes and fibroblasts by increasing the expression of fibronectin, filaggrin, Ki-67, Bcl-2, inhibin beta A, and Cyclin D1. It also acted as an agonist of the adenosine A2A receptor and induced the phosphorylation of focal adhesion kinase, adenosine triphosphate-dependent tyrosine kinase, and mitogen-activated protein kinase. This activated signal transduction, thereby regenerating skin cells and strengthening the barrier. These results were not only observed in skin cells but also in an artificial skin model (KeraSkin^TM^). The use of plant-derived PDRN instead of animal-derived PDRN can promote animal welfare and environmental sustainability. Furthermore, Panax PDRN can potentially be a new plant-derived PDRN (PhytoPDRN) that may be utilized in the treatment of various skin diseases.

## 1. Introduction

Polydeoxyribonucleotide (PDRN) is a linear sperm DNA polymer commonly obtained from the gonads of salmon trout. It is composed of a mixture of deoxyribonucleotides with a chain length of 80–2200 base pairs and a molecular weight between 50 and 1500 kDa. The nucleotides include adenosine, which has been found to have various pharmacological effects by binding it to adenosine receptors [1,2,3]. Adenosine has a wide range of physiological and pathological functions, which are mediated by four different receptors known as A1, A2A, A2B, and A3. Among these, signal transduction through the A2A receptor is known to be a major mechanism involved in regulating inflammation, ischemia, cell proliferation, and angiogenesis [4,5]. Polydeoxyribonucleotide is typically an agonist of the A2A receptor, which regulates various intracellular actions [4,6,7].

Polydeoxyribonucleotide has been found to be effective in promoting wound healing [4,8], tissue regeneration, and inflammation reduction, as well as in the regulation of cytokine signaling pathways involved in collagen production [9,10]. In addition, PDRN is known to enhance corneal epithelial regeneration and relieve pain and disability in patients with rotator cuff disease [11,12,13]. Moreover, it improves wound closure and promotes re-epithelialization in patients with refractory diabetic foot ulcers and is particularly effective in promoting the regeneration of skin wounds [14]. Polydeoxyribonucleotide stimulates wound healing by acting as a growth promoter and chemokine, thereby promoting the proliferation of epithelial cells in the wound [6,15,16].

The root of Korean ginseng (Panax ginseng C.A. Meyer) has been used in the traditional treatment of various diseases in East Asia for over 2000 years [17]. It is well known for its excellent anti-inflammatory, anti-tumorigenic, antioxidant, and anti-aging properties [18,19,20,21]. Korean ginseng root contains not only polysaccharides and polyacetylene acid but also ginsenosides (Rb1, Rb2, Rc, Rd, and Rg1) with pharmacological effects, which have been investigated in many studies [22]. In addition to ginsenosides, it contains proteins, nucleic acids, lipids, and metabolites that exert various other effects. However, the mechanism and efficacy of these compounds have yet to be extensively studied [17]. Several pharmacological studies have investigated the anti-aging properties of ginseng extract and ginsenoside. Ginsenoside Rb1 is known to prevent cell death caused by ultraviolet (UV) radiation and increase the anti-aging effect of the skin by stimulating the production of type I collagen [23,24]. Similarly, ginsenoside F1 has been found to inhibit the aging of human dermal fibroblasts caused by UVB and prevent the death of human keratinocyte (HaCaT) cells induced by UVB [25].

In this study, we aimed to confirm that PDRN extracted from Panax ginseng has similar effects to that extracted from salmon testes on skin cell regeneration and the enhancement of the skin barrier. Panax PDRN purified from suspension-cultivated Panax ginseng adventitious roots may serve as a plant-derived PDRN (PhytoPDRN) that can enhance skin cell regeneration and strengthen the skin barrier through similar physiological processes to those used by animal-sourced PDRN.

## 2. Results

### 2.1. Isolation and Purification of Panax PDRN from Panax Ginseng Adventitious Roots

**Figure 1:**
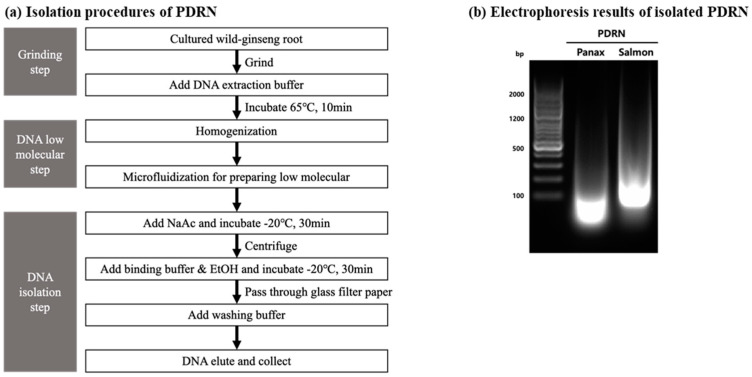
Isolation and purification of Panax PDRN from adventitious roots of Panax ginseng using microfluidization. (**a**) Panax PDRN was isolated and purified from cultured cells mass-produced by plant tissue culture technology using an ultra-high-pressure extraction method. Low-molecular Panax PDRN can be obtained via hydrolysis, excluding chemicals. (**b**) The DNA size of purified Panax PDRN was compared with that of salmon PDRN through gel electrophoresis.

Panax PDRN was extracted and purified from the adventitious roots of cultured wild ginseng. The use of chemical processes such as acid hydrolysis to lower the molecular weight of the extracted PDRN poses a risk of DNA denaturation and safety concerns due to the chemical residue. To avoid these risks, we used a microfluidizer that employs an ultra-high-pressure physical crushing method to isolate the PDRN, which avoided the risk of chemical denaturation and also extracted PDRN possessing improved pharmacological effects (Figure 1a).

To verify the similarity in physical characteristics, namely, DNA size, between purified Panax PDRN and animal-derived PDRN (salmon PDRN), we performed agarose gel electrophoresis. The results showed that Panax PDRN had a DNA size slightly smaller than 100 base pairs, whereas salmon PDRN had a size slightly larger than 100 base pairs. Although there was a small difference in DNA sizes, there was no significant difference in the patterns of the two PDRNs (Figure 1b).

### 2.2. Panax PDRN Promotes Cell Proliferation

**Figure 2:**
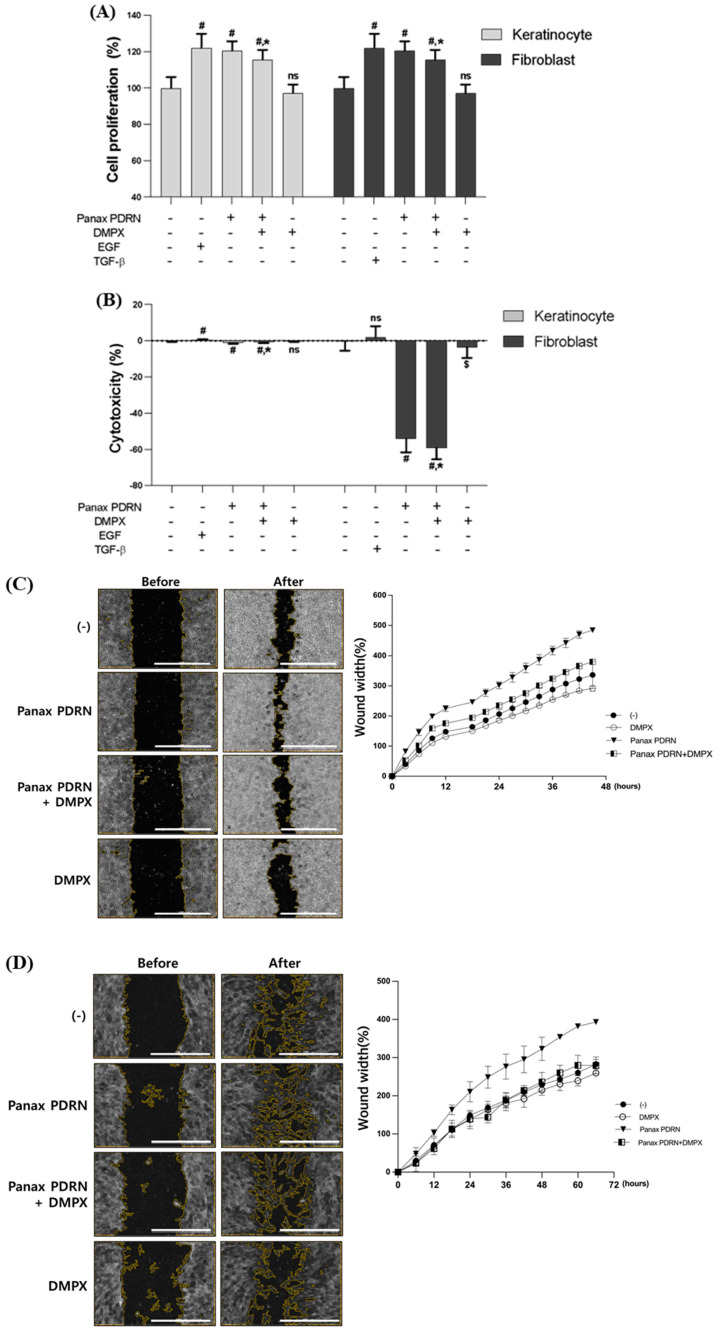

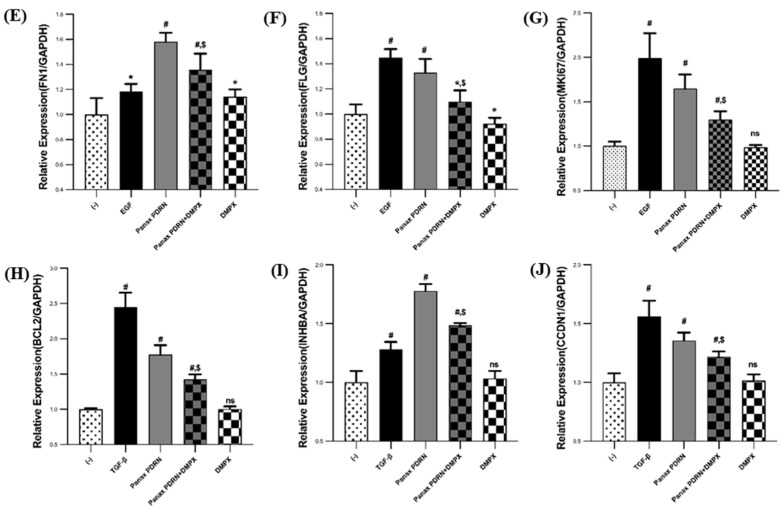
Analysis of cell proliferation and regeneration-promoting effects of Panax PDRN on keratinocytes and fibroblasts. (**A**) Cell proliferation and (**B**) cytotoxicity were analyzed for Panax PDRN in keratinocytes (HaCaT) and fibroblasts (HDF). (**C**,**D**) A wound-healing assay was performed to confirm cell proliferation and regeneration ability in HaCaT and HDF (scale bar represents 1000 μm). (**E**–**J**) The expression of each gene related to cell proliferation and regeneration (fibronectin, filaggrin, Ki-67, Bcl-2, inhibin A, and cyclin D1) was confirmed via qRT-PCR in HaCaT and HDF. mRNA levels were normalized to GAPDH expression. Expression levels for each gene are indicated as fold-changes compared with the control levels. ^#^ *p* < 0.01 compared with basal levels; * *p* < 0.05; ^$^ *p* < 0.01 compared with Panax PDRN levels; ns means statistically not significant; DMPX: 3,7-Dimethyl-1-propargylxanthine.

To investigate the effect of Panax PDRN on cell proliferation, we evaluated its efficacy on HaCaT and HDF cells using the CCK-8 (cholecystokinin octapeptide) method. Treatment with Panax PDRN resulted in a 20% increase in cell proliferation in both HaCaT and HDF cells. In order to investigate whether the efficacy of Panax PDRN is related to the A2A receptor pathway, similar to that reported for animal-derived PDRN, the A2A receptor inhibitor 3,7-Dimethyl-1-propargylxanthine (DMPX) was additionally treated. The cells treated with Panax PDRN and DMPX showed 7.5% reduced proliferation compared with cells treated with Panax PDRN alone in both HaCaT and HDF cells. Panax PDRN also showed similar a induction of cell proliferation in EGF and TGF-β treatments (Figure 2A). EGF and TGF-β were treated as positive controls for keratinocytes and dermal fibroblasts, respectively. Retinoic acid was also used as a positive control for skin barrier function improvement. Additionally, an LDH (lactate dehydrogenase) assay revealed that Panax PDRN was not cytotoxic (Figure 2B).

To verify that Panax PDRN promotes cell proliferation, a wound-healing assay was performed on both cell types. Panax PDRN increased regeneration by 30.6% in HaCaT cells and by 28.3% in HDF cells. When treated with DMPX, regeneration induced by Panax PDRN was reduced in both cell types (Figure 2C,D). This increase in cell proliferation may be linked to an increase in specific gene expression. Panax PDRN increased the expression of fibronectin (FN1), filaggrin (FLG), and Ki-67 (MKI67) in keratinocytes (Figure 2E–G) and B cell lymphoma 2 (BCL2), inhibin beta A (INHBA), and cyclin D1 (CCDN1) in fibroblasts (Figure 2H–J).

### 2.3. Panax PDRN Activates the A2A Receptor and Regulates Cell Proliferation through the FAK-AKT-Mitogen-Activated Protein Kinase (MAPK) Signaling Pathway

**Figure 3:**
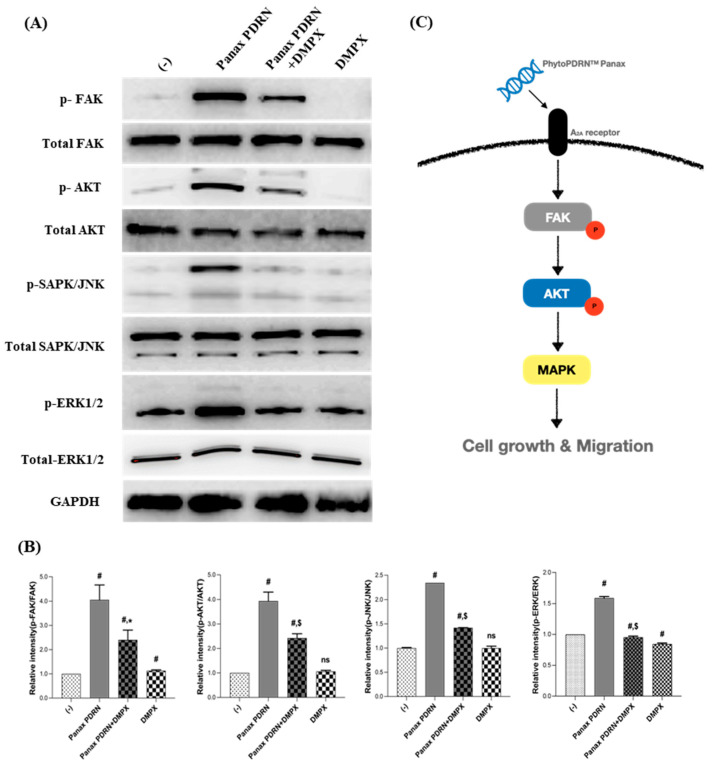
Effect of Panax PDRN on FAK-KT-MAPK signaling in keratinocytes. (**A**) FAK and AKT phosphorylation and MAPK activation were assessed via Western blot analysis, and (**B**) the quantitative results are shown. (**C**) Panax PDRN can activate a signaling pathway that induces cell proliferation through the A2A receptor. ^#^ *p* < 0.01 compared with basal levels; * *p* < 0.05; ^$^ *p* < 0.01 compared with Panax PDRN levels; ns means statistically not significant.

To investigate whether Panax PDRN activates the A2A receptor signaling pathways that are associated with cell proliferation and barrier improvement similar to the activation caused by animal-derived PDRN, we treated HaCaT cells with Panax PDRN at a 5% concentration. The treatment led to a 4.05-fold increase in protein phosphorylation for FAK, a 3.96-fold increase for AKT, a 2.34-fold increase for SAPK/JNK, and a 1.58-fold increase for ERK1/2. However, when treated with DMPX, an A2A receptor inhibitor, protein phosphorylation was reduced (Figure 3A,B). These results suggest that Panax PDRN can activate a signaling pathway that induces cell proliferation through the A2A receptor (Figure 3C).

### 2.4. Analysis of Wound Healing Effect of Panax PDRN in 3D Skin Model

**Figure 4:**
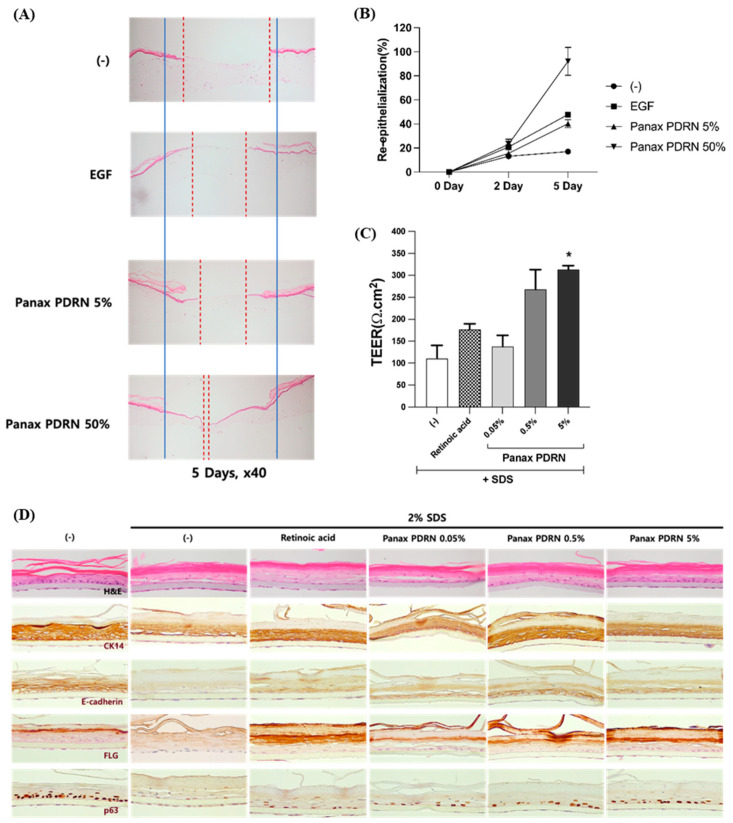
Skin tissue regeneration and barrier function improvement effect of Panax PDRN in a 3D skin model. (**A**,**B**) Analysis of the skin regeneration ability in the 3D skin model using the wound-healing assay when treated with 5% or 50% Panax PDRN. (**C**) After treatment with Panax PDRN, the recovery of the damaged skin barrier was confirmed through TEER measurement and (**D**) immunostaining in the tissue. * *p* < 0.05 compared with basal levels. Red dash line: non epithelialized area; blue solid line: initial wound area.

In this study, we used a human skin model to evaluate the wound-healing efficacy of Panax PDRN. The results showed that when Panax PDRN was applied to the wound on the human skin model, there was an increase in re-epithelialization on the second and fifth days compared with the untreated group. On the second day, the re-epithelialization rate was 15.5% in the 5% PDRN-application group and 23.2% in the 50% PDRN-application group, whereas it was 13.2% in the untreated group and 20.8% in the EGF-application group. On the fifth day, the re-epithelialization rate was 40.3% in the 5% PDRN-application group and 92.0% in the 50% PDRN-application group, whereas it was 17.0% in the untreated group and 47.9% in the EGF-application group. Notably, the 50% PDRN-application group showed similar re-epithelialization rates to EGF on the second day and a significant increase in re-epithelialization over time (Figure 4B).

### 2.5. Restoration of Skin Barrier Function

We evaluated the ability of Panax PDRN to improve the barrier function of skin tissue using a human skin model damaged by SDS, an anionic surfactant, by measuring the trans-epidermal electrical resistance (TEER) and analyzing the expression of skin differentiation markers using immunohistochemistry. Treatment with various concentrations of Panax PDRN led to a rise in TEER values that correlated with the dose used, with the highest increase observed at a concentration of 0.5% (Figure 4C). Additionally, the expression levels of certain skin differentiation markers were found to be higher in the Panax PDRN-treated group than in the control group damaged by SDS, as determined via immunohistochemistry analysis. Specifically, the levels of filaggrin expression rose as the concentration of Panax PDRN increased. Furthermore, the expression patterns of E-cadherin and p63 revealed that Panax PDRN was more effective in restoring barrier function than retinoic acid (Figure 4D).

## 3. Discussion

Over the last 30 years, many researchers worked to create more effective methods of isolating and purifying nucleic acids and proteins from plants, as these substances are becoming increasingly important in medicine and plant science. In this study, we used a microfluidizer, which utilizes an ultra-high-pressure physical crushing method, to obtain PDRN from plants and found that samples containing PDRN were effective in promoting skin-wound healing and improving the skin barrier. Polydeoxyribonucleotide is a type of DNA polymer with a molecular weight between 50 and 1500 KDa; unlike regular DNA, it does not carry genetic information but exhibits pharmacological activities. Polydeoxyribonucleotide is typically extracted and purified from the sperm DNA of chum salmon or salmon trout [4]. Polydeoxyribonucleotide exhibits various physiological effects, among which is the ability to act as an agonist of the A2A receptor, which is a signal transducer involved in skin regeneration. By activating this receptor, PDRN promotes the secretion of various growth factors, enhances capillary formation through vascular endothelial growth factor, improves blood circulation, and acts as an anti-inflammatory agent [5]. Polydeoxyribonucleotide can effectively promote skin regeneration and help heal wounds such as burns with minimal side effects. Furthermore, studies have demonstrated that PDRN can quickly promote tissue regeneration and decrease the time needed for wound healing [26,27]. Notably, PDRN extracted from mass-produced plants using plant tissue culture technology can contribute to environmental protection and the improvement of animal welfare by reducing indiscriminate fishing or hunting while still providing benefits similar to animal-derived PDRN.

We obtained Panax PDRN by cultivating and mass-purifying it from the adventitious roots of Korean ginseng, a plant with a long history of medicinal use attributed to its various pharmacological functions. In the past, chemical methods such as acid hydrolysis have been used to purify or reduce the size of PDRN, but these methods have the potential to denature its DNA and leave behind chemical residues. In this study, we purified and low-molecularized Panax PDRN using a physical method involving ultra-high-pressure grinding.

Cell proliferation and migration, key factors in wound healing, can be enhanced by treatments with animal-derived PDRN [4]. Focal adhesion kinase plays a significant role in cell morphogenesis and migration by regulating F-actin polymerization and focal adhesion turnover [28,29,30]. The activity of FAK increases the activity of AKT and MAPK downstream signaling pathways [1,28]. Furthermore, MAPK signaling, including FAK and JNK, has been shown to promote cell proliferation and migration [28,31]. As demonstrated in Figure 3, Panax PDRN acts as an agonist of the A2A receptor and increases the phosphorylation of FAK, AKT, and MAPK (JNK and ERK1/2), thereby promoting cell proliferation and migration (Figure 2A–D). These processes contribute to wound healing and the improvement of skin barrier function. To confirm the wound-healing efficacy of Panax PDRN, we used a 3D skin model (KeraSkin-FT^TM^; Biosolution Co. Ltd., Seoul, Korea) This skin model, when exposed to air for several days, forms a basal, prickle, and granular layer, as well as a stratum corneum. Its characteristics are similar to human skin tissue, making it suitable for various efficacy evaluations [32,33]. Wound healing occurs in four stages: hemostasis, inflammation, proliferation, and maturation. The main process that leads to healing in the proliferation stage is the recovery of the wound surface and the formation of granulation tissue [34]. Based on these properties, it was shown that Panax PDRN can promote wound healing (Figure 4A,B). Additionally, an experiment was conducted on KeraSkin-FT^TM^ to confirm the effect of PDRN on skin barrier improvement and wound healing. Tight junctions, which are one of the types of cell-cell bonds, are developed in the epidermal layer, creating a physicochemical barrier. The degree of electrical resistance across the cell layer can be measured using TEER, and the higher the barrier ability, the higher the TEER value [35]. Cytokeratin 14 (CK14), E-cadherin, p63, and FLG are factors related to skin barrier function. The CK14 protein forms the skeleton of epidermal cells and is expressed in the basal and prickle layers. E-cadherin is an intercellular junction protein expressed in the cell membrane as a cell-cell contract marker, and p63 is a protein that regulates cell proliferation and is mainly expressed in the intracellular nucleus. The FLG protein is an essential factor in maintaining the homeostasis of the epidermis layer. It acts as an adhesive within cells and is converted into a natural moisturizing factor that affects skin moisturizing. The FLG protein is expressed from the granular layer to the stratum corneum [36,37,38,39]. Retinoic acid, which is used as a control, affects the skin barrier function by regulating the expression of claudin-1 and 4, which are proteins that control tight junctions [40]. Panax PDRN not only increased the TEER value of the tissue model damaged by SDS (Figure 4C) but also restored the expression of filaggrin, E-cadherin, p63, and CK14. Therefore, we could verify the efficacy of Panax PDRN in improving skin barrier function (Figure 4D).

Although the effects of Panax PDRN concerning various concentrations were not assessed in vitro, the efficacy of PDRN in various concentrations was analyzed in the 3D human tissue model. The 3D human tissue model showed a dose-dependent increase due to the Panax PDRN treatment. The phenotypic protein expressions in tissue-like skin cell layers can better reflect the in vivo-like physiology.

## 4. Materials and Methods

### 4.1. Isolation of PDRN from Panax Ginseng Adventitious Roots

Panax ginseng adventitious roots were grown in a culture media consisting of Schenk and Hildebrandt medium, 0.01 mg/L indole butyric acid, and 3% sucrose. After a month, the adventitious roots were harvested to extract PDRN. The extracted PDRN was purified from the adventitious roots using a modified version of the filter-paper-based spin column method [41] (Figure 1A).

Briefly, the Panax ginseng adventitious roots were finely crushed using liquid nitrogen, and 10 g of the adventitious root powder was combined with 50 g of DNA extraction buffer containing 0.1% *w*/*w* sodium dodecyl sulfate (SDS), 0.5 M NaCl, and 0.2 M Tris-HCl at pH 7.5. The mixture was incubated at 65 °C for 10 min. To break down the cell wall, the mixture was homogenized for 10 min. The lysates were then further homogenized at high pressure using a microfluidizer (PICOMAX, Seoul, Korea) to produce a low-molecular-weight form. Thereafter, the lysate was clarified via centrifugation at 7000 rpm for 20 min. Subsequently, sodium acetate (1/10th volume of 3 M) was added to the supernatant. After being incubated at −20 °C for 30 min, the sample was centrifuged at 7000 rpm for 20 min. To bind the DNA to filter paper, a half-volume of binding buffer (4 M guanidine hydrochloride in 80% ethanol) was added to the sample, which was incubated at −20 °C for 30 min. Thereafter, the sample was passed through a filtration apparatus with glass fiber paper to bind the PDRN. To wash the filter paper, 100 mL of washing buffer (10 mM NaCl and 10 mM Tris-HCl in 80% ethanol (pH 6.5)) was added. The filter paper was washed again with 100 mL of 95% ethanol. After washing, it was left to dry at room temperature for 10 min. Thereafter, deionized water (10 mL) was added to the filter paper containing bound PDRN and filtered through a vacuum filtration apparatus. The eluted PDRN was collected and quantified. The quality and quantity of the DNA were measured using a Nanodrop UV spectrometer (BioTek, El Segundo, CA, USA).

### 4.2. Characterization of PDRN Isolated from Panax Ginseng Adventitious Roots

To compare the molecular size of PDRN extracted from P. ginseng and salmon, DNA agarose gel electrophoresis was performed. The PDRN was run on a 1.5% (*w*/*v*) agarose gel with a 100 bp molecular marker (GeneAll Biotechnology, Seoul, Korea). The nucleic acid-stained DNA fluorescence was visualized and captured using a Gel Imaging Analysis System (BIO-RAD, Hercules, CA, USA).

### 4.3. Cell Culture, Cell Proliferation, Cytotoxicity, and Quantitative Reverse Transcription PCR (qRT-PCR)

Human dermal fibroblasts (HDF) were obtained from ATCC (Manassas, VA, USA), and HaCaT cells were obtained from the Cell Lines Service (Eppelheim, Germany). The HDF cells were grown in Human Fibroblast Expansion Basal Medium (ThermoFisher, Waltham, MA, USA) with penicillin-streptomycin and Low Serum Growth Supplement (ThermoFisher). The HaCaT cells were grown in Dulbecco’s modified Eagle’s medium with penicillin-streptomycin and 10% fetal bovine serum (Capricon Scientific, Ebsdorfergrund, Germany). Both cell types were grown in an incubator (ThermoFisher) at 37 °C and 5% CO_2_. Cell proliferation and toxicity tests were conducted using a serum-free medium. The epidermal growth factor (EGF; R&D Systems, Minneapolis, MN, USA) and transforming growth factor-beta (TGF-β; R&D Systems) were used as positive controls for the proliferation of HaCaT and HDF, respectively. The treatment concentration was equally 10 ng/mL. The cells were also treated with Panax PDRN at a concentration of 5% and 3,7-Dimethyl-1-proparglyxanthine (DMPX; Sigma-Aldrich, St. Louis, MO, USA), an A2A receptor inhibitor, at a concentration of 10 nM. After culturing the cells for 72 h, cell proliferation was analyzed using Cell Counting Kit-8 (Dojindo, Kumamoto, Japan), and cytotoxicity was analyzed using Cytotoxicity Lactate Dehydrogenase (LDH) Assay Kit-WST (Dojindo). The analysis was carried out as per the manufacturer’s instructions.

Total RNA was extracted using the TaKaRa MiniBEST Universal RNA Extraction Kit (TaKaRa Bio, Shiga, Japan), and complementary DNA (cDNA) was synthesized from the extracted RNA using the amfiRivert cDNA Synthesis Platinum Master Mix (GenDEPOT, Katy, TX, USA) according to the manufacturer’s instructions. Gene expression analysis using qRT-PCR was performed with TB Green^®^ Premix Ex Taq™ (TaKaRa Bio) and CronoSTAR™ 96 Real-Time PCR System (TaKaRa Bio) according to the manufacturer’s instructions using the following primer sequences:

FN1 fwd: 5′-GCCAGTCCTACAACCAGTATTC-3′, rev: 5′-CTCGGGAATCTTCTCTGTCAG-3′, FLG for: 5′-CGGGTTTAGACACTCTCAGCA-3′

3′, rev: 5′-GCCACATAAACCTGGGTCCTT-3′, MKI67 for: 5′-ATGCCAGCACCAGAGGAAATTG-3′, rev: 5′-TCTCTTCATGATGACCACGGGT-3′, BCL2 for: 5′-TCTTTGAGTTCGGTGGGGTCAT

3′, rev: 5′-GGTTCAGGTACTCAGTCATCCA-3′, INHBA for: 5′-CCACTCAACAGTCATCAACCAC-3′, rev: 5′-ACAACATGGACATGGGTCTCAG-3′, CCDN1 for: 5′-TTCATTTCCAATCCGCCCTCCA-3′, rev: 5′-CACACTTGATCACTCTGGAGAG-3′. GAPDH for: 5′-CATCAAGAAGGTGGTGAAGCAGG-3′, rev: 5′-AGTGGTCGTTGAGGGCAATGC-3′.

### 4.4. Western Blotting

The HaCaT cells were harvested 48 h after being treated with 5% Panax PDRN and 10 nM DMPX. Total protein was extracted from the harvested cells using CytoBuster™ Protein Extraction Reagent (EMD Millipore Corp, Bedford, MA, USA), and thereafter, Xpert Phosphatase Inhibitor Cocktail Solution (GenDEPOT) and Xpert Protease Inhibitor Cocktail Solution (GenDEPOT) were added to it. The protein samples were loaded onto Precast Lumi-Gels, 4–20% (LumiNano, Seoul, Korea), and run on SDS-polyacrylamide gels, which were then transferred to polyvinylidene difluoride membranes (GenDEPOT). The blots were subsequently treated with various antibodies to detect the presence or absence of protein bands. The antibodies and their concentrations used were as follows: phosphorylated focal adhesion kinase (FAK); stress-activated protein kinase (SAPK)/Jun amino-terminal kinases (JNK); extracellular signal-regulated kinase (ERK)1/2 (dilution 1:1000; Cell Signaling Technology, MA, USA); phosphorylated protein kinase B (AKT) (dilution 1:1000; R&D Systems); total FAK, SAPK/JNK, and ERK1/2 (dilution 1:1000; Cell Signaling Technology); glyceraldehyde-3-phosphate dehydrogenase (GAPDH; dilution 1:5000; Cell Signaling Technology); and total AKT (dilution 1:1000; R&D Systems). All protein bands were quantified using the Image J software (V1.47t; National Institute of Health, Bethesda, MD, USA), which revealed significant differences between the N.C. and treatments. Data are represented as mean ± SD.

### 4.5. Production of Artificial 3D Skin Model

KeraSkin^TM^ (Biosolution, Seoul, Korea) is an artificial skin model composed of normal human skin keratinocytes, and KeraSkin-FT^TM^ (Biosolution) is a full-thickness artificial skin model composed of human normal keratinocytes, human normal fibroblasts, and a collagen matrix. To prepare KeraSkin^TM^, human keratinocytes were seeded on 12 mm Millicell^®^ (Millipore, USA) and proliferation was induced until confluent for 5 days of submerged culture. After that, epithelial differentiation was induced via air-liquid interface culture for 7 days with 3T3 feeder layers. To prepare KeraSkin-FT^TM^, a dermal layer was reconstructed by mixing HDF with type I collagen, and a full-thickness human skin model was made by seeding human skin keratinocytes onto the dermal layer. The subsequent culture was performed in the same manner as for KeraSkin^TM^.

### 4.6. Wound Healing Assay

After culturing HaCaT and HDF cells in a 24-well plate, wounds were created at regular width. After wound generation, 5% Panax PDRN, 10 nM DMPX (Sigma-Aldrich), 10 ng/mL EGF, and 10 ng/mL TGF-β were added to each well. Cell regeneration was then analyzed using the Lionheart FX Automated Microscope (BioTek, CA, USA), a real-time imaging analysis instrument.

To evaluate the effectiveness of Panax PDRN on the artificial skin model, a wound was created on the surface of KeraSkin-FT^TM^. Briefly, a straight line was cut on the surface of KeraSkin-FT^TM^ using two surgical blades placed 1 mm apart, and both ends were trimmed with surgical scissors. Thereafter, 50 μL of 10 ng/mL EGF with 5% or 50% Panax PDRN was applied to the wound surface. The cells were then grown for five days in a 37 °C, 5% CO_2_ incubator (Sanyo, Osaka, Japan).

### 4.7. Evaluation of Skin Barrier-Improving Ability and Measurement of Transepithelial Electrical Resistance (TEER)

To disrupt the skin barrier of KeraSkin^TM^, 2% sodium dodecyl sulfate (Sigma-Aldrich) was applied to the surface of KeraSkin^TM^ for 3 min and then washed with Dulbecco’s phosphate-buffered saline (Capricorn, Ebsdorfergrund, Germany). Subsequently, 50 μL of 10 uM retinoic acid (Sigma-Aldrich) and 0.05%, 0.5%, or 5% Panax PDRN were applied, following which, the cells were incubated (Sanyo) at 37 °C and 5% CO_2_ for 48 h.

To measure the TEER of KeraSkin^TM^, the Panax PDRN application was removed from the surface of KeraSkin^TM^ via washing. The tissue was transferred to a new 6-well plate, and TEER was measured using an ERS-2 instrument (EMD Millipore).

### 4.8. Hematoxylin and Eosin (H&E) Staining

For histopathological analysis, tissues previously treated with test samples during the skin barrier improvement evaluation were fixed with 10% formalin for 3 h at room temperature and embedded in paraffin. Thereafter, paraffin specimens were sectioned on a glass slide with a thickness of 4 μm; deparaffinized with xylene and 100%, 90%, 80%, and 70% ethanol (in a consecutive manner); stained with H&E solution (EMS, Harrisburg, PA, USA); washed with sterile distilled water; dehydrated with xylene and 95% and 100% ethanol (in a consecutive manner); and finally mounted.

### 4.9. Immunohistochemistry

To analyze protein expression in the tissue, the tissue treated with Panax PDRN was fixed with 10% formalin for 3 h at room temperature and embedded in paraffin through a general tissue specimen preparation process. Thereafter, paraffin specimens were sectioned on a glass slide with a thickness of 4 μm; deparaffinized with xylene (Sigma-Aldrich) and 100%, 90%, 80%, and 70% ethanol (in a consecutive manner); treated with 0.3% Triton X-100 (Sigma-Aldrich) and 0.3% H_2_O_2_ (Sigma-Aldrich) solutions; and pretreated with sodium citrate heat inuced epitope retrieval, pH 6.0. The following primary antibodies were used: filaggrin (MyBioSource, San Diego, CA, USA), cytokeratin 14 (Abcam, Cambridge, UK), p63 (Abcam), and E-cadherin (Abcam). The samples were then stained using the Vector ABC kit (Vector Labs, Newark, CA, USA) and the DAB substrate kit (Vector Labs). Thereafter, the tissue was dehydrated with xylene and 95% and 100% ethanol (in a consecutive manner) and finally mounted.

### 4.10. Statistical Analyses

All results are presented as the mean ± standard deviation. Statistical significance was determined by using GraphPad Prism 9 (GraphPad Software, Inc., CA, USA). All experiments were performed in triplicate and repeated three times.

## 5. Conclusions

This study concludes that PhytoPDRN, a PDRN isolated from plants using tissue culture technology, has the ability to promote skin regeneration and protect the skin barrier, making it a viable alternative to animal-derived PDRN. This study specifically focuses on the efficacy of PhytoPDRN obtained from Korean ginseng adventitious roots, but in the future, research will be focused on PDRN isolated from other plants. Furthermore, we aim to establish PhytoPDRN as a suitable replacement for animal-derived PDRN through comparison and equivalence experiments.

## Data Availability

The data presented in this study are available upon request from the corresponding author. The data are not publicly available because of the confidentiality of the relevant company, Biosolution Co., Ltd.

## References

[1] Ko I.G., Jin J.J., Hwang L., Kim S.H., Kim C.J., Jeon J.W., Chung J.Y., Han J.H. (2021). Adenosine A2A receptor agonist polydeoxyribonucleotide ameliorates short-term memory impairment by suppressing cerebral ischemia-induced inflammation via MAPK pathway. PLoS ONE.

[2] Tonello G., Daglio M., Zaccarelli N., Sottofattori E., Mazzei M., Balbi A. (1996). Characterization and quantitation of the active polynucleotide fraction (PDRN) from human placenta, a tissue repair stimulating agent. J. Pharm. Biomed. Anal..

[3] Zhou Y., Zeng X., Li G., Yang Q., Xu J., Zhang M., Mao X., Cao Y., Wang L., Xu Y. (2019). Inactivation of endothelial adenosine A(2A) receptors protects mice from cerebral ischaemia-induced brain injury. Br. J. Pharmacol..

[4] Hwang K.H., Kim J.H., Park E.Y., Cha S.K. (2018). An effective range of polydeoxyribonucleotides is critical for wound healing quality. Mol. Med. Rep..

[5] Colangelo M.T., Galli C., Guizzardi S. (2020). The effects of polydeoxyribonucleotide on wound healing and tissue regeneration: A systematic review of the literature. Regen. Med..

[6] Veronesi F., Dallari D., Sabbioni G., Carubbi C., Martini L., Fini M. (2017). Polydeoxyribonucleotides (PDRNs) From Skin to Musculoskeletal Tissue Regeneration via Adenosine A(2A) Receptor Involvement. J. Cell Physiol..

[7] Thellung S., Florio T., Maragliano A., Cattarini G., Schettini G. (1999). Polydeoxyribonucleotides enhance the proliferation of human skin fibroblasts: Involvement of A2 purinergic receptor subtypes. Life Sci..

[8] Melani A., Pugliese A.M., Pedata F. (2014). Adenosine receptors in cerebral ischemia. Int. Rev. Neurobiol..

[9] Altavilla D., Squadrito F., Polito F., Irrera N., Calo M., Lo Cascio P., Galeano M., La Cava L., Minutoli L., Marini H. (2011). Activation of adenosine A2A receptors restores the altered cell-cycle machinery during impaired wound healing in genetically diabetic mice. Surgery.

[10] Squadrito F., Bitto A., Irrera N., Pizzino G., Pallio G., Minutoli L., Altavilla D. (2017). Pharmacological Activity and Clinical Use of PDRN. Front. Pharmacol..

[11] Yoon Y.C., Lee D.H., Lee M.Y., Yoon S.H. (2017). Polydeoxyribonucleotide Injection in the Treatment of Chronic Supraspinatus Tendinopathy: A Case-Controlled, Retrospective, Comparative Study With 6-Month Follow-Up. Arch. Phys. Med. Rehabil..

[12] Lee S.H., Zheng Z., Kang J.S., Kim D.Y., Oh S.H., Cho S.B. (2015). Therapeutic efficacy of autologous platelet-rich plasma and polydeoxyribonucleotide on female pattern hair loss. Wound Repair. Regen..

[13] Valdatta L., Thione A., Mortarino C., Buoro M., Tuinder S. (2004). Evaluation of the efficacy of polydeoxyribonucleotides in the healing process of autologous skin graft donor sites: A pilot study. Curr. Med. Res. Opin..

[14] Squadrito F., Bitto A., Altavilla D., Arcoraci V., De Caridi G., De Feo M.E., Corrao S., Pallio G., Sterrantino C., Minutoli L. (2014). The effect of PDRN, an adenosine receptor A2A agonist, on the healing of chronic diabetic foot ulcers: Results of a clinical trial. J. Clin. Endocrinol. Metab..

[15] Buffoli B., Favero G., Borsani E., Boninsegna R., Sancassani G., Labanca M., Rezzani R., Nocini P.F., Albanese M., Rodella L.F. (2017). Sodium-DNA for Bone Tissue Regeneration: An Experimental Study in Rat Calvaria. Biomed. Res. Int..

[16] Noh T.K., Chung B.Y., Kim S.Y., Lee M.H., Kim M.J., Youn C.S., Lee M.W., Chang S.E. (2016). Novel Anti-Melanogenesis Properties of Polydeoxyribonucleotide, a Popular Wound Healing Booster. Int. J. Mol. Sci..

[17] Cho E.G., Choi S.Y., Kim H., Choi E.J., Lee E.J., Park P.J., Ko J., Kim K.P., Baek H.S. (2021). Panax ginseng-Derived Extracellular Vesicles Facilitate Anti-Senescence Effects in Human Skin Cells: An Eco-Friendly and Sustainable Way to Use Ginseng Substances. Cells.

[18] Hwang E., Park S.Y., Yin C.S., Kim H.T., Kim Y.M., Yi T.H. (2017). Antiaging effects of the mixture of Panax ginseng and Crataegus pinnatifida in human dermal fibroblasts and healthy human skin. J. Ginseng Res..

[19] Kang T.H., Park H.M., Kim Y.B., Kim H., Kim N., Do J.H., Kang C., Cho Y., Kim S.Y. (2009). Effects of red ginseng extract on UVB irradiation-induced skin aging in hairless mice. J. Ethnopharmacol..

[20] Hong C.E., Lyu S.Y. (2011). Anti-inflammatory and Anti-oxidative Effects of Korean Red Ginseng Extract in Human Keratinocytes. Immune Netw..

[21] Park H.J., Kim D.H., Park S.J., Kim J.M., Ryu J.H. (2012). Ginseng in traditional herbal prescriptions. J. Ginseng Res..

[22] Lu J.M., Yao Q., Chen C. (2009). Ginseng compounds: An update on their molecular mechanisms and medical applications. Curr. Vasc. Pharmacol..

[23] Kwok H.H., Yue P.Y., Mak N.K., Wong R.N. (2012). Ginsenoside Rb(1) induces type I collagen expression through peroxisome proliferator-activated receptor-delta. Biochem. Pharmacol..

[24] Cai B.X., Jin S.L., Luo D., Lin X.F., Gao J. (2009). Ginsenoside Rb1 suppresses ultraviolet radiation-induced apoptosis by inducing DNA repair. Biol. Pharm. Bull..

[25] Lee E.H., Cho S.Y., Kim S.J., Shin E.S., Chang H.K., Kim D.H., Yeom M.H., Woe K.S., Lee J., Sim Y.C. (2003). Ginsenoside F1 protects human HaCaT keratinocytes from ultraviolet-B-induced apoptosis by maintaining constant levels of Bcl-2. J. Investig. Dermatol..

[26] Shin D.Y., Park J.U., Choi M.H., Kim S., Kim H.E., Jeong S.H. (2020). Polydeoxyribonucleotide-delivering therapeutic hydrogel for diabetic wound healing. Sci. Rep..

[27] Kwon T.R., Han S.W., Kim J.H., Lee B.C., Kim J.M., Hong J.Y., Kim B.J. (2019). Polydeoxyribonucleotides Improve Diabetic Wound Healing in Mouse Animal Model for Experimental Validation. Ann. Dermatol..

[28] Singkhorn S., Tantisira M.H., Tanasawet S., Hutamekalin P., Wongtawatchai T., Sukketsiri W. (2018). Induction of keratinocyte migration by ECa 233 is mediated through FAK/Akt, ERK, and p38 MAPK signaling. Phytother. Res..

[29] Zhao X., Guan J.L. (2011). Focal adhesion kinase and its signaling pathways in cell migration and angiogenesis. Adv. Drug Deliv. Rev..

[30] Serrels B., Serrels A., Brunton V.G., Holt M., McLean G.W., Gray C.H., Jones G.E., Frame M.C. (2007). Focal adhesion kinase controls actin assembly via a FERM-mediated interaction with the Arp2/3 complex. Nat. Cell Biol..

[31] Sulzmaier F.J., Jean C., Schlaepfer D.D. (2014). FAK in cancer: Mechanistic findings and clinical applications. Nat. Rev. Cancer.

[32] Song D., Park H., Lee S.H., Kim M.J., Kim E.J., Lim K.M. (2017). PAL-12, a new anti-aging hexa-peptoid, inhibits UVB-induced photoaging in human dermal fibroblasts and 3D reconstructed human full skin model, Keraskin-FT. Arch. Dermatol. Res..

[33] Buskermolen J.K., Reijnders C.M., Spiekstra S.W., Steinberg T., Kleverlaan C.J., Feilzer A.J., Bakker A.D., Gibbs S. (2016). Development of a Full-Thickness Human Gingiva Equivalent Constructed from Immortalized Keratinocytes and Fibroblasts. Tissue Eng. Part. C Methods.

[34] Reinke J.M., Sorg H. (2012). Wound repair and regeneration. Eur. Surg. Res..

[35] Srinivasan B., Kolli A.R., Esch M.B., Abaci H.E., Shuler M.L., Hickman J.J. (2015). TEER measurement techniques for in vitro barrier model systems. J. Lab. Autom..

[36] Kezic S., Jakasa I. (2016). Filaggrin and Skin Barrier Function. Curr. Probl. Dermatol..

[37] Guo Y., Redmond C.J., Leacock K.A., Brovkina M.V., Ji S., Jaskula-Ranga V., Coulombe P.A. (2020). Keratin 14-dependent disulfides regulate epidermal homeostasis and barrier function via 14-3-3sigma and YAP1. eLife.

[38] Van den Bossche J., Van Ginderachter J.A. (2013). E-cadherin: From epithelial glue to immunological regulator. Eur. J. Immunol..

[39] Kim S., Choi I.F., Quante J.R., Zhang L., Roop D.R., Koster M.I. (2009). p63 directly induces expression of Alox12, a regulator of epidermal barrier formation. Exp. Dermatol..

[40] Li J., Li Q., Geng S. (2019). All-trans retinoic acid alters the expression of the tight junction proteins Claudin-1 and -4 and epidermal barrier function-associated genes in the epidermis. Int. J. Mol. Med..

[41] Shi R., Lewis R.S., Panthee D.R. (2018). Filter paper-based spin column method for cost-efficient DNA or RNA purification. PLoS ONE.

